# Inhibiting NHEJ in HNSCC cell lines by the ligase IV inhibitor SCR130 has limited radiosensitizing effects

**DOI:** 10.1038/s41598-025-03159-5

**Published:** 2025-05-22

**Authors:** Laura S. Hildebrand, Tina Jost, Marion Schindler, Anja Derer, Gregor Fuhrmann, Rainer Fietkau, Luitpold V. Distel

**Affiliations:** 1 Department of Radiation Oncology, Universitätsklinikum Erlangen, Friedrich-Alexander-Universität Erlangen-Nürnberg (FAU), Universitätsstraße 27, 91054 Erlangen, Germany; 2 Comprehensive Cancer Center Erlangen-EMN (CCC ER-EMN), Erlangen, Germany; 3 Department of Radiation Oncology, Translational Radiobiology, Universitätsklinikum Erlangen, Friedrich-Alexander-Universität Erlangen-Nürnberg (FAU), Universitätsstraße 27, 91054 Erlangen, Germany; 4 Department of Biology, Pharmaceutical Biology, Friedrich-Alexander-Universität Erlangen-Nürnberg (FAU), Staudtstraße 5, 91058 Erlangen, Germany

**Keywords:** Ligase IV, SCR130, Head and neck squamous cell carcinoma, DNA damage response, Radiation sensitivity, Ionizing radiation, Radiotherapy, Targeted therapies, Head and neck cancer

## Abstract

Radiotherapy (RT) is a relevant treatment for head and neck squamous cell carcinoma (HNSCC) patients but radioresistance, which depends on DNA damage response (DDR), restrains outcome. Therefore, manipulating DDR by small molecule inhibitors (SMI) is a promising treatment option. The main DNA double strand break (DSB) repair mechanisms in healthy mammalian cells are homologous recombination (HR) and non-homologous end joining (NHEJ). It is known that HR is already often impaired in tumors because of cancerous transitions. Therefore, additionally inhibiting NHEJ is a possibility to specifically target tumor cells and spare healthy tissue, which has the alternative DSB repair mechanism available. We treated HNSCC and healthy fibroblast cell lines with 30 µM of the ligase IV inhibitor SCR130 and a single dose of 2 Gy (Gy) ionizing radiation (IR) to investigate the inhibitor’s radiosensitizing effect. In short, the effect of SCR130 in combination with IR on cell death, clonogenicity, and DNA damage is limited and highly cell line specific. Nevertheless, SCR130 increases the number of cells in G0/G1 phase concomitant with gained p21 expression consistently. We suggest that SCR130 in combination with IR has anti-proliferative effects, but an escape of the cells by upregulation of ligase IV resulting from the treatment is possible.

**Supplementary Information:**

The online version contains supplementary material available at 10.1038/s41598-025-03159-5.

## Introduction

Head and neck squamous cell carcinoma (HNSCC) is a very heterogenous group of tumor diseases affecting the upper respiratory tract and swallowing organs^1^. HNSCCs are relevant worldwide but risk and prognosis differ depending on country and demography; highest incidence rates appear in South and Southeast Asia and more men than women are affected^1^. Risk factors are mainly - on the one hand - infections with high risk human papilloma virus (HPV); on the other hand, tobacco and alcohol abuse, summarized as HPV-negative cancers^2^. In the last decades, it was not possible to improve treatment of HNSCC patients significantly so survival rates stagnate^2^ and optimized therapies are urgently needed. Established treatment options for HNSCC patients are surgery, chemotherapy, and radiotherapy (RT)^2^. Advanced multimodal treatment schemes also include immunotherapy; however, the outcome is variable^3,4^. Clinical trials identifying responder populations and optimized immunomodulation are currently ongoing^3,4^.

Problematically, radioresistance of HNSCC is a common issue, which has to be overcome to increase patients’ prognosis^2,5^. Radioresistance is elevated proportional to the tumor cells’ ability to handle ionizing radiation (IR) -induced DNA damage^5^. However, DNA damage response (DDR) is often pathologically altered in cancer cells, which can reduce the efficacy of radiation in the tumor^5^. To address the radioresistance of HNSCCs we inhibited their DDR with the ligase IV inhibitor SCR130 and additionally induced DNA damage by IR. We hypothesize that this combined treatment should radiosensitize the cells and increase the efficacy of the IR.

There is clinical evidence that HPV-negative HNSCCs are less radiosensitive than HPV-positive ones, resulting in poorer prognosis and the need of improved treatment schemes^6–8^. Biological differences are not understood completely, but the reasons are probably multifactorial; it is conceivable that DDR, tumor microenvironment, and immune reactions to the viral proteins influence treatment response^6^. To overcome treatment resistance, novel therapeutics and useful combination with available options are essential^9,10^. Small molecule inhibitors (SMI) are a promising advanced treatment option to open the therapeutic window by specifically targeting tumor cells^11^. For RT, SMI that inhibit DDR are of special interest because they have the potential to radiosensitize tumor cells^12^.

**Fig. 1:**
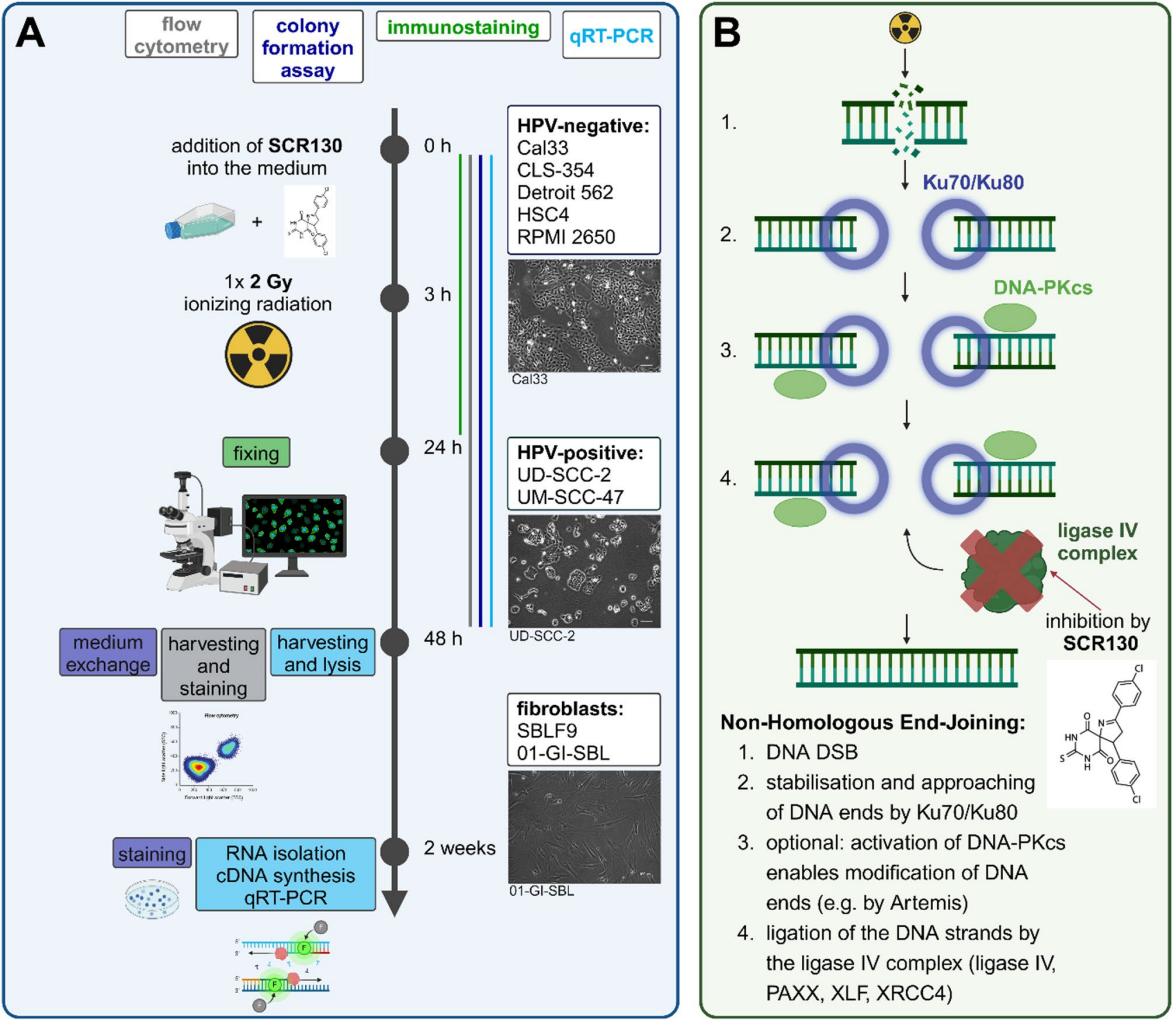
Overview of the experimental workflow. (**A**) Timeline of treatment and measurement of the four analytical methods flow cytometry, colony formation assay, immunostaining, and qRT-PCR used to determine the effect of the ligase IV inhibitor SCR130 on head and neck squamous cell carcinoma (HNSCC) cell lines. SCR130 was always added to the medium 3 h prior to irradiation with 2 Gy (Gy) ionizing radiation (IR). The treatment time was 48 h except for immunostaining where it was reduced because of rapid DNA repair. Representative images of one HPV-negative (Cal33), HPV-positive (UDSCC-2), and fibroblast (01-GI-SBL) cell lines are shown^29^. (**B**) Principle of DNA double strand break (DSB) repair by non-homologous end joining (NHEJ): Process and most important DNA repair proteins of NHEJ and its interaction with SCR130; adapted from^14,15^, Created in BioRender. Distel, L. (2024) BioRender.com/k76p847.

Healthy mammalian cells have several repair mechanisms available, but IR-induced DNA double strand breaks (DSB) are repaired mainly by homologous recombination (HR) and non-homologous end joining (NHEJ), depending on the cell cycle state and the complexity of the DNA damage^13^. For NHEJ DNA ends are stabilized by dimeric Ku70/Ku80 protein complex (Ku70/Ku80) followed by optional modifications of the DNA ends^14,15^. In the very last step, the ligase IV complex - consisting of ligase IV, X-ray repair cross-complementing protein 4 (XRCC4), XRCC4-like factor (XLF), and paralog of XRCC4 and XLF (PAXX) - rejoins the loose DNA ends (Fig. 1B)^14,15^. The advantage of NHEJ is that it is very fast and efficient, which is essential for genomic integrity, but it is also error-prone^13,16^. In contrast, HR requires a homologous sequence of the damaged region of DNA, usually the sister chromatid; this enables error-free repair and prevents genetic instability^16^. Consequently, HR is limited to S and G2 phase of the cell cycle, whereas NHEJ is permanently available^16^.

We focused on NHEJ because it is possible throughout the whole cell cycle and therefore very efficient and essential for cell survival^13^. An additional advantage of inhibiting NHEJ is that HR in tumor cells is often non-functional or only partially functional due to mutations^17–19^; this means that tumors are at a distinct disadvantage compared to normal tissue cells when NHEJ is blocked and their HR is not functional.

We hypothesize inhibiting NHEJ should enable specifically targeting tumor and sparing normal tissue cells. As mentioned, an essential enzyme for the NHEJ is ligase IV. There are very few cases of patients with the so-called ligase IV syndrome, which is defined by genetic changes in the ligase IV encoding gene^20^. Consequently, NHEJ is impaired in those patients causing - among other symptoms – increased radiation sensitivity^20^. Therefore, we chose ligase IV as the target for our SMI treatment; it is one of the most essential DNA repair proteins in NHEJ and it is known that ligase IV deficiency leads to radiation sensitivity in cell lines^21^ and in patients^20^.

The relevance of combining SMI of DNA DSB repair with IR was shown in several studies for example by Faulhaber and Jost et al. who inhibited DNA-dependent protein kinase catalytic subunit (DNA-PKcs)/mechanistic target of rapamycin kinase (mTOR), ataxia telangiectasia mutated (ATM), and ataxia telangiectasia and Rad3-related protein (ATR) and showed increased efficacy by a combined treatment with IR in cancer cell lines *in vitro*^22^. There is also a wide variety of clinical studies ongoing combining IR with DNA repair inhibition summarized by Monge-Cadet et al.^23^. Peposertib for example inhibits NHEJ by targeting repair-relevant DNA-PKcs, which has been studied in patients in multiple clinical trials^23,24^.

Taken together ligase IV is a promising target protein for a SMI in combination with IR to increase radiation sensitivity in HNSCC cell lines. Already in 2012, Srivastava et al. presented the ligase IV inhibitor SCR7, which increased unrepaired DNA DSB and cytotoxicity and decreased proliferation in cancer cell lines in a ligase IV baseline dependent manner^25^. Moreover, they showed response in three of four tested cancer mouse models resulting from SCR7 treatment and elevated radiation sensitivity in a combined treatment of SCR7 and IR^25^. Nevertheless, a study from Greco et al. from 2016 questions the effectiveness and specificity of the SCR7 molecule^26^. Therefore, in 2020 Ray et al. described several molecules derived from SCR7 structure and suggested SCR130 being the most promising ligase IV inhibitor and potential candidate for radiosensitizing cancer cells^27^. There is a lack of knowledge if SCR130 could be clinically relevant for treating HNSCCs and especially the radioresistant HPV-negative ones, which we addressed in a preclinical setting.

We hypothesize that ligase IV inhibition by a specific and efficient SMI is a promising strategy to radiosensitize HNSCC cell lines and therefore serve as a treatment option for radioresistant HPV-negative cancer patients^6–8^. Therefore, we characterized the effect of SCR130 treatment combined with IR on HPV-positive and HPV-negative HNSCC cell lines.

Additionally, we included healthy fibroblasts to calculate adverse effects on healthy tissue. In our study, we investigated cell death, cell cycle distribution, clonogenicity, DNA damage, and the underlying mechanisms on mRNA level.

## Methods

### Cell cultivation

Seven different commercially available HNSCC cell lines namely Cal33, CLS-354, Detroit 562, HSC4, RPMI 2650, UD-SCC-2, UM-SCC-47, and two healthy fibroblast cell lines SBLF9 and 01-GI-SBL were cultivated at 37 °C and 5% CO_2_ in a humidified atmosphere continuously. UD-SCC-2 and UM-SCC-47 are HPV-positive in contrast to all the other HNSCC cell lines. Cal33, HSC4, UD-SCC-2, and UM-SCC-47 were provided by Dr. Thorsten Rieckmann (University Medical Centre Hamburg-Eppendorf, Germany). CLS-354, Detroit 562, RPMI 2650 were purchased from CLS Cell Lines Service GmbH (Eppelheim, Germany). SBLF9 and 01-GI-SBL were obtained by biopsy from healthy male donors’ skin or oral mucosa respectively and fibroblasts were cultivated subsequently as described previously^28^. Donors signed the informed consent and Ethics Committee of the medical faculty of the Friedrich-Alexander-Universität Erlangen-Nürnberg (204_17 BC) agreed on August 18, 2017.

SBLF9 were cultivated in F12 medium (Gibco, Waltham, USA) supplemented with 15% fetal bovine serum (FBS) (FBS Superior, Sigma-Aldrich, Darmstadt, Germany), 2% non-essential amino acids (100x, Bio&SELL GmbH, Feucht, Germany), 1% penicillin-streptomycin (Gibco, Waltham, USA). All the other cell lines were cultivated in Dulbecco’s modified eagle medium (PANBiotech GmbH, Aidenbach, Germany) with 10% FBS and 1% penicillin-streptomycin. Cells were split (0.5% Trypsin-ethylenediaminetetraacetic acid [EDTA], Gibco, Waltham, USA) every third or fourth day in an appropriate ratio before reaching complete confluence. Mycoplasma status was determined regularly using Mycoplasma Detection Kit (InvivoGen Europe, Toulouse, France).

### Inhibitor and irradiation

SCR130 (catalogue number: S9775, molecular weight: 418.3, Selleck Chemicals LLC, Houston, USA) was diluted in dimethyl sulfoxide (DMSO) (Carl Roth GmbH + Co. KG, Karlsruhe, Germany) according to its molecular weight reaching a concentration of 10 mM. This stock solution was added directly into the medium to a final concentration of 15 µM or 30 µM. Inhibitor stock solution was thawed freshly before every use. Irradiation with 2 Gy (Gy) IR was carried out using an ISOVOLT Titan X-ray generator (GE Inspection Technologies, Ahrensburg, Germany). For the standard treatment, SCR130 stock solution was added 3 h prior to irradiation. DMSO control was treated with the same volume DMSO as used for SCR130 stock solution to serve as vehicle control; 2 Gy control only received IR (Fig. 1A).

### Flow cytometry for cell death and cell cycle analysis

Cells were seeded in cell culture flasks and incubated 24 h before the medium was changed to serum reduced medium (2%). Immediately, SCR130 stock solution or a corresponding volume of DMSO was added and 3 h afterwards cells were irradiated as described above. Cell number was adjusted to reach a maximum of 80% confluence after three days. After 48 h of inhibitor treatment, cells were harvested. Therefore, cells, supernatant, and washing solution (Dulbecco’s phosphate buffered saline [DPBS], Gibco, Waltham, USA) were collected. Cell pellet was divided equally for cell death and cell cycle analysis.

For cell cycle analysis 1 mL 2% medium and 10 mL cold 70% ethanol (Otto Fischar GmbH & Co. KG, Saarbrücken, Germany) were added to the pellet. Cells were fixed at least overnight at 4 °C. Before the measurement, 1 mL Ringer solution (Fresenius Kabi AG, Bad Homburg, Germany) and 3 µL Hoechst 33342 (10 mg/mL, Molecular Probes, Waltham, USA) were added to the cell pellet and incubated 1 h at 4 °C. Afterwards, the staining solution was removed immediately by centrifugation and cells were resuspended in 100–150 µL Ringer solution. Forward and sideward scatter light and Hoechst signal were measured with Cytoflex S (Beckman Coulter GmbH, Krefeld, Germany).

Immediately after harvesting, the staining for cell death analysis was carried out. The cell pellet was resuspended in 200 µL Ringer solution and 10 µL allophycocyanin (APC) Annexin V/7Aminoactinomycin D (7AAD) (BD Biosciences, Franklin Lakes, USA) mix (1:1). After 30 min of incubation at 4 °C, the cell pellet was resuspended in 100–150 µL Ringer solution and forward and sideward scatter light, Annexin V, and 7AAD signal were measured. Flow cytometry data were analyzed using Kaluza Analysis Version 2.1.00003.20057.

### Colony formation assay

Cells were seeded in Petri dishes (60 mm diameter) ensuring good separation of the single cells from each other and incubated 17 ± 1 h. Afterwards, SCR130 stock solution was added into the medium to reach the final concentration. After 3 h of incubation, half of the Petri dishes were irradiated with 2 Gy IR. After 48 h of inhibitor treatment, the medium was exchanged with fresh medium to remove the inhibitor.

Cells were incubated until colonies of at least 50 cells were formed in every condition (7–14 days, cell line dependent). Finally, colonies were stained with Wright’s eosin methylene blue solution (Carl Roth GmbH + Co. KG, Karlsruhe, Germany) for 30 min, images were acquired, and colonies were quantified using Biomas Software (V3.0 7/2012, Erlangen, Germany). Plating efficiency (PE) is defined as the proportion of colonies to the seeded cell number. Surviving fraction (SF) is calculated dividing the PE of treated conditions by the PE of the DMSO control.

### Immunostaining

Cells were seeded in silicone chambers (flexiPERM^®^, SARSTEDT AG & Co. KG, Nümbrecht, Germany) placed on glass slides or directly on glass slides to reach maximum 90% confluence. After 48 h of incubation, medium was exchanged to medium containing inhibitor with the final concentration (for untreated controls only fresh medium). Three hours later, cells were irradiated with 2 Gy IR.

After 24 h of inhibitor treatment, cells were washed with DPBS once, fixed, and permeabilized with 4% / 0.1% formaldehyde / Triton X-100 in DPBS (formaldehyde solution 37%, Carl Roth GmbH + Co. KG, Karlsruhe, Germany / Triton™ X-100, Sigma-Aldrich, Darmstadt, Germany) for 15 min, and finally washed for five min with 1x Tris-buffered saline (TBS) three times. Cells were blocked in blocking solution (1% bovine serum albumin [BSA] [SERVA Electrophoresis GmbH, Heidelberg, Germany], 10% FBS in DPBS) minimum over night at 4 °C.

For primary antibody staining, slides were washed with 1x TBS as described above and then incubated with primary antibody mixture [phospho-histone H2A.X (Ser 139), rabbit, 1:400, Cell Signaling Technology, Inc., Danvers, USA; DNA-ligase IV, mouse, 1:50, Santa Cruz Biotechnology, Inc., Dallas, USA in 1% BSA in 1x TBS] over night at 4 °C in a humidified atmosphere. For secondary antibody staining, slides were washed with 1x TBS again and incubated with secondary antibody mix (Alexa Fluor 488 donkey anti-rabbit; Alexa Fluor 555 donkey anti-mouse; Thermo Fisher Scientific Inc., Waltham, USA, 1:200 in 1% BSA in 1x TBS) 90 min at room temperature in a humidified atmosphere. After another washing step, 0.3 mg/L 2-(4-Amidinophenyl)-1*H*-indole-6-carboxamidine (DAPI) in 4x saline sodium citrate buffer/Tween was added for one minute. Glass slides were washed in distilled water and dried. Finally, staining was mounted with Vectashield^®^ antifade mounting medium with DAPI (Vector Laboratories, Inc., Burlingame, USA) and coverslips.

Additionally, different time points and a higher dose were tested exemplary in Cal33. Therefore, fixation was carried out 4, 21, and 48 h after irradiation and 0 Gy, 2 Gy, and 4 Gy IR were compared.

Images were captured using Axio Imager.Z2 fluorescence microscope (Carl Zeiss AG, Oberkochen, Germany) with imaging software Metafer 4 (V 3.10.7, MetaSystems Hard and Software GmbH, Altlussheim, Germany) and analyzed using Biomas Software (V3.0 7/2012, Erlangen, Germany). Foci were counted automatically when the difference between the brightness of the focus and the background was higher than the defined threshold.

### qRT-PCR

Cells were seeded into six-well cell culture plates (two wells per biological replicate) 24 h prior to inhibitor treatment. The seeded cell number was adjusted to reach 80% confluence for harvesting. Cells were treated with inhibitor or DMSO as described above and irradiated with a single dose of 2 Gy IR 3 h afterwards.

After 48 h of inhibitor treatment, cells were harvested and lyzed in 1 mL trizol per sample (Invitrogen, Thermo Fisher Scientific Inc., Waltham, USA). Phenol-chloroform extraction was used to isolate RNA. In short, after chloroform addition and centrifugation the resulting upper aqueous phase was collected and RNA was precipitated with isopropanol.

After three washing steps with ethanol, RNA pellet was dried and diluted in water. Genomic DNA (gDNA) was digested at 37 °C for 30 min (DNase I, catalogue number: EN0521, Thermo Fisher Scientific Inc., Waltham, USA) before 1–2 µg RNA were transcribed to complementary DNA (cDNA) according to the manufacturer’s instructions (High-Capacity cDNA Reverse Transcription Kit, catalogue number: 4368813, Thermo Fisher Scientific Inc., Waltham, USA). Controls without reverse transcriptase were included (NRT). Finally, quantitative real-time polymerase chain reaction (qRT-PCR) was done using DyNAmo ColorFlash SYBR Green qPCR Kit (catalogue number: F416XL, Thermo Fisher Scientific Inc., Waltham, USA) and primer pairs obtained from Bio-Rad (Table 1) according to the manufacturer’s instructions.

Relative gene expression was calculated using two housekeeper genes (*ACTB*, *HMBS*) relative to DMSO control. For quality control NRT, no template control (NTC, water instead of sample), the difference between technical duplicates, and housekeeper stability were consulted.

**Table 1:** Primer pairs used for quantitative real-time polymerase chain reaction (qRT-PCR) analysis. *ACTB* and *HMBS* served as housekeeper genes. All primer pairs were obtained from Bio-Rad laboratories GmbH, Feldkirchen, Germany and can be identified with their unique assay ID. Protein names according to www.genecards.org.

Gene	Protein	Unique assay ID (Bio-Rad)
*ACTB*	Actin beta	qHsaCED0036269
*HMBS*	Hydroxymethylbilane synthase	qHsaCID0038839
*ABCB1*	ATP binding cassette subfamily B member 1 (alternative: P-glycoprotein)	qHsaCED0056970
*ABCG2*	ATP binding cassette subfamily G member 2 (JR blood group)	qHsaCED0057063
*CDKN1A*	Cyclin dependent kinase inhibitor 1 A (alternative: p21)	qHsaCID0014498
*CDKN2A*	Cyclin dependent kinase inhibitor 2 A (alternative: p16)	qHsaCED0056722
*CHEK1*	Checkpoint kinase 1	qHsaCID0037755
*LIG4*	DNA ligase IV	qHsaCED0047042
*RAD51*	RAD51 recombinase	qHsaCID0007576

### Flow cytometry to determine C12FDG signal

5-Dodecanoylaminofluorescein Di-β-D-Galactopyranosid (C12FDG) is a marker associated with senescence; to identify the induction of C12FDG signal by the treatment, one T25 flask per condition was seeded with 50,000 cells. The next day, medium was exchanged to fresh standard cultivation medium and cells were treated according to the standard treatment scheme as described above. At day ten after seeding, cells were harvested collecting supernatant, washing solution, and cell suspension as described for cell death and cell cycle analysis. The pellet was resuspended in 400 µL standard cultivation medium and 4 µL 10 µM Bafilomycin A1 (Streptomyces griseus, catalogue number: 196000, Merck KGaA, Darmstadt, Germany) were added to the pellet of every flask and an incubation of 30 min at 37 °C followed. Afterwards, 4 µL 10 mg/mL Hoechst 33342 were added, followed by another 30-minute incubation. Finally, 0.5 µL 20 mM C12FDG (catalogue number: D2893, Invitrogen, Thermo Fisher Scientific Inc., Waltham, USA) were added and cells were incubated at 37 °C for one hour at 37 °C. After centrifugation, the pellet was resuspended in 200 µL Ringer solution and 10 µL APC Annexin V/7AAD mix (1:1) and was incubated for 30 min in the fridge. Lastly, cell pellets were resuspended in 150 µL Ringer solution and Hoechst, C12FDG, Annexin, and 7AAD signals were measured at the Cytoflex S.

### Optimization of the treatment scheme

To increase the effect of SCR130, the standard protocol - namely treatment of cells with 30 µM SCR130 diluted in DMSO 3 h prior to irradiation with 2 Gy IR - was modified and cell death and cell cycle analysis were repeated.

For the treatment with liposomes, the same time line was used but instead of SCR130 in DMSO the inhibitor was delivered in liposomes. The same volume was pipetted as in the standard treatment (1:333 in 5 mL per T25 cell culture flask). Unloaded liposomes served as control.

For the reverse treatment, application of IR and inhibitor treatment was changed, meaning the inhibitor addition was carried out 3 h after dose application. The incubation time of 24 h after seeding and the treatment time of 48 h were identical. Moreover, the dose of IR was increased.

### Preparation of liposomes

For the preparation of SCR130 loaded liposomes, thin film hydration was used as described by Drost et al.^30^. 3192 µL 18:1 1,2-dioleoyl-sn-glycero-3-phospho-(1’-rac-glycerol) (DOPG), 3952 µL 18:1 1’,3’-bis[1,2-dioleoyl-sn-glycero-3-phospho]-glycerol (Cardiolipin) (both 25 mg/mL in chloroform from Avanti Polar Lipids, Inc; Part of Croda International Plc, Alabaster, USA), and 441 µL 25 mg/mL cholesterol in chloroform (both Merck KGaA, Darmstadt, Germany) were mixed. 200 µL of this solution were poured in Amber glass vials. Moreover, a 10 mM solution of SCR130 in ethyl acetate was prepared and half of the vials were loaded additionally with 10 µL of the SCR130 solution; the other half served as unloaded control.

The lipid films were dried in the fume hood overnight and stored at -20 °C afterwards until further steps. Each lipid film was diluted in 1 mL MilliQ water and heated up to 72 °C. The vials were vortexed for 40 min repeatedly. Afterwards, extrusion through a membrane (Extruder Set with Holder/Heating Block and Hamilton Syringes; Whatman^®^ membrane filters mixed cellulose ester, pore size 0.2 μm, diameter 47 mm; filter support; all from Merck KGaA, Darmstadt, Germany) followed and liposomes were collected.

To separate liposomes from free SCR130, a size exclusion chromatography with sepharose CL-2B was carried out and fractions of 1 mL were collected. A nanoparticle tracking analyzer (NTA) was used to determine the fraction with the highest liposome concentration and their size (Table 2).

**Table 2:** Concentration and diameter of SCR130 loaded and unloaded liposomes measured by nanoparticle tracking analyzer (NTA).

	Concentration [particles/mL]	Diameter [nm]
Unloaded liposomes	4.1 × 10^10^	84.4-473.4
Liposomes loaded with SCR130	3.2 × 10^12^	116.4

### Graphs and statistics

Graphs were generated using GraphPad Prism 9.5.1 (GraphPad Software, San Diego, USA), Microsoft Excel 2019 (Microsoft Corporation, Redmond, USA), and BioRender (Science Suite Inc., Toronto, Canada). Statistically analysis was carried out using GraphPad Prism.

## Results

Our goal was to increase the efficacy of RT by combining it with a SMI that inhibits DNA DSB repair and therefore elevates the IR-induced DNA damage. The ligase IV inhibitor SCR130 seems suitable due to its interaction with NHEJ. We adapted the clinical setting using a standard single dose of 2 Gy IR and compared the combined treatment (30 µM SCR130 + 2 Gy) with IR alone. We used a variety of assays to study the effect of the ligase IV inhibitor SCR130 combined with IR on seven HNSCC and two healthy fibroblast cell lines.

### SCR130 combined with IR potentially induces necrotic cell death

**Fig. 2:**
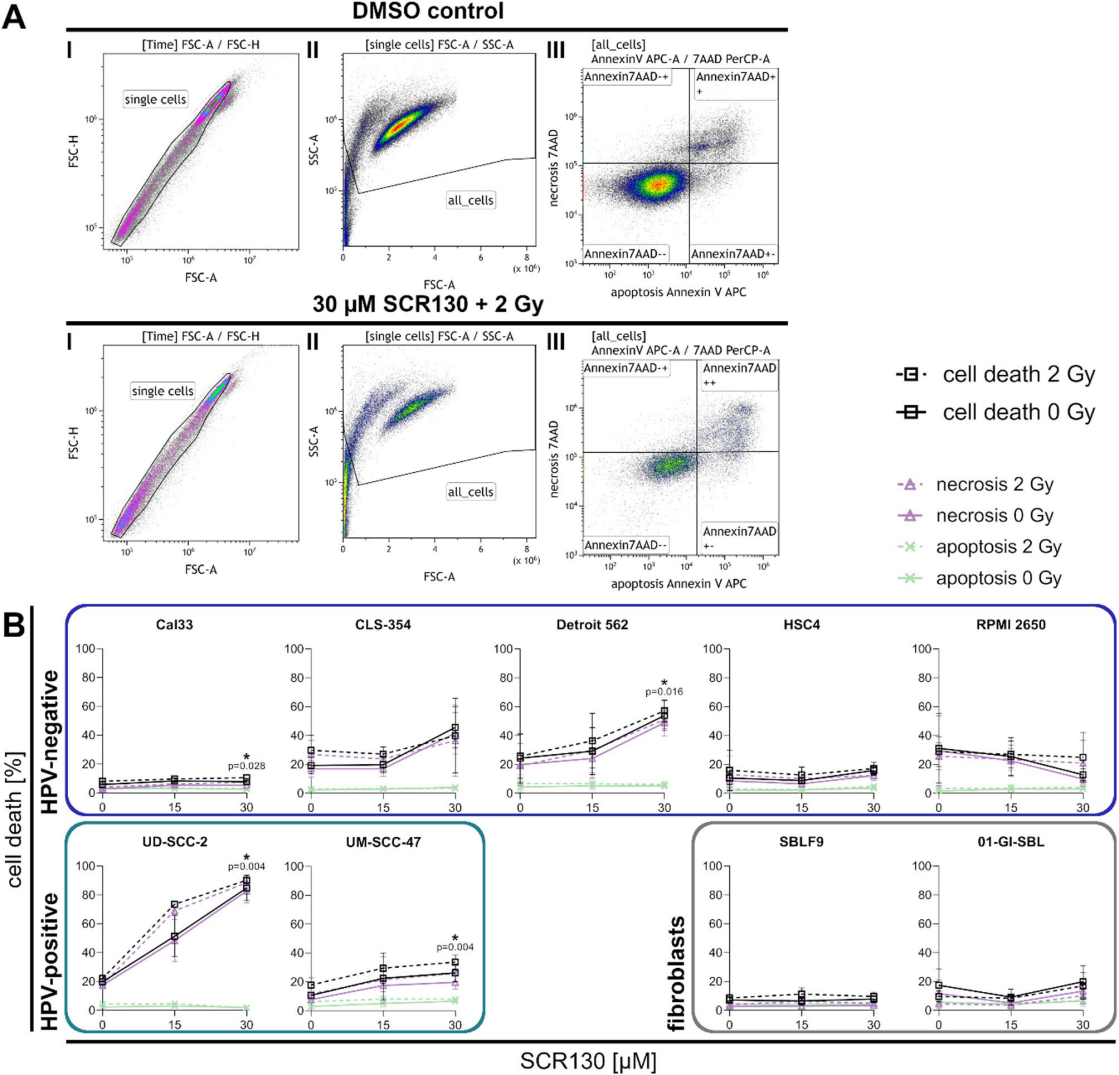
Flow cytometry analysis of cell death. (**A**) Gating strategy: Exemplary flow cytometry results of UM-SCC-47 treated with vehicle control (dimethyl sulfoxide, DMSO) or combined treatment (30 µM SCR130 + 2 Gy). First, doublets (I) and cell debris (II) were excluded. Afterwards, the amount of living cells (Annexin7AAD--), apoptotic cells (Annexin7AAD+-), and necrotic cells (Annexin7AAD++) was determined (III). (**B**) Cell death analysis: apoptotic (Annexin, green), necrotic (7AAD, purple) and sum of dead cells (apoptotic + necrotic, black) in different human papilloma virus (HPV) -negative and HPV-positive HNSCC cell lines and healthy fibroblasts after treatment. Cells were treated with 15 or 30 µM SCR130; half of the samples additionally received 2 Gy IR 3 h afterwards. Vehicle control was treated with the same volume DMSO as used for 30 µM SCR130 (0 Gy). Statistically significant differences were calculated between 2 Gy and 30 µM SCR130 + 2 Gy (sum of dead cells) by two-tailed Mann-Whitney test to evaluate the radiosensitizing effect of SCR130. Each value represents mean ± SD (*n* ≥ 3).

To investigate the induction of cell death, cells were stained with APC Annexin V and 7AAD. The gating strategy allowed excluding doublets and cell debris by forward and side scatter and to identify Annexin V and 7AAD negative cells (Annexin7AAD--) as living cells. Apoptotic cells were defined as Annexin7AAD+- and necrotic cells as Annexin7AAD++ (Fig. 2A). In Cal33, Detroit 562, UD-SCC-2, and UM-SCC-47 a significant induction of cell death (*p* = 0.004–0.028, two-tailed Mann-Whitney test) was detectable comparing IR with the combined treatment (black dashed line), which indicates an increased efficacy of IR in combination with SCR130. Also, CLS-354 tended to increase cell death. The apoptotic cell population did not increase after IR, SCR130, or the combined treatment in either HNSCC cell lines or fibroblasts (green lines). Also, in the fibroblasts, HSC4, and RPMI 2650 there was no increase in the necrotic fraction detectable whereas in the other cell lines necrosis increased (purple lines). In HSC4 and RPMI 2650, neither SCR130 nor the combination induced any cell death at all, so there was no improvement detectable compared to IR alone (Fig. 2B).

### SCR130 combined with IR induces a G0/G1 block in tumor cells

**Fig. 3:**
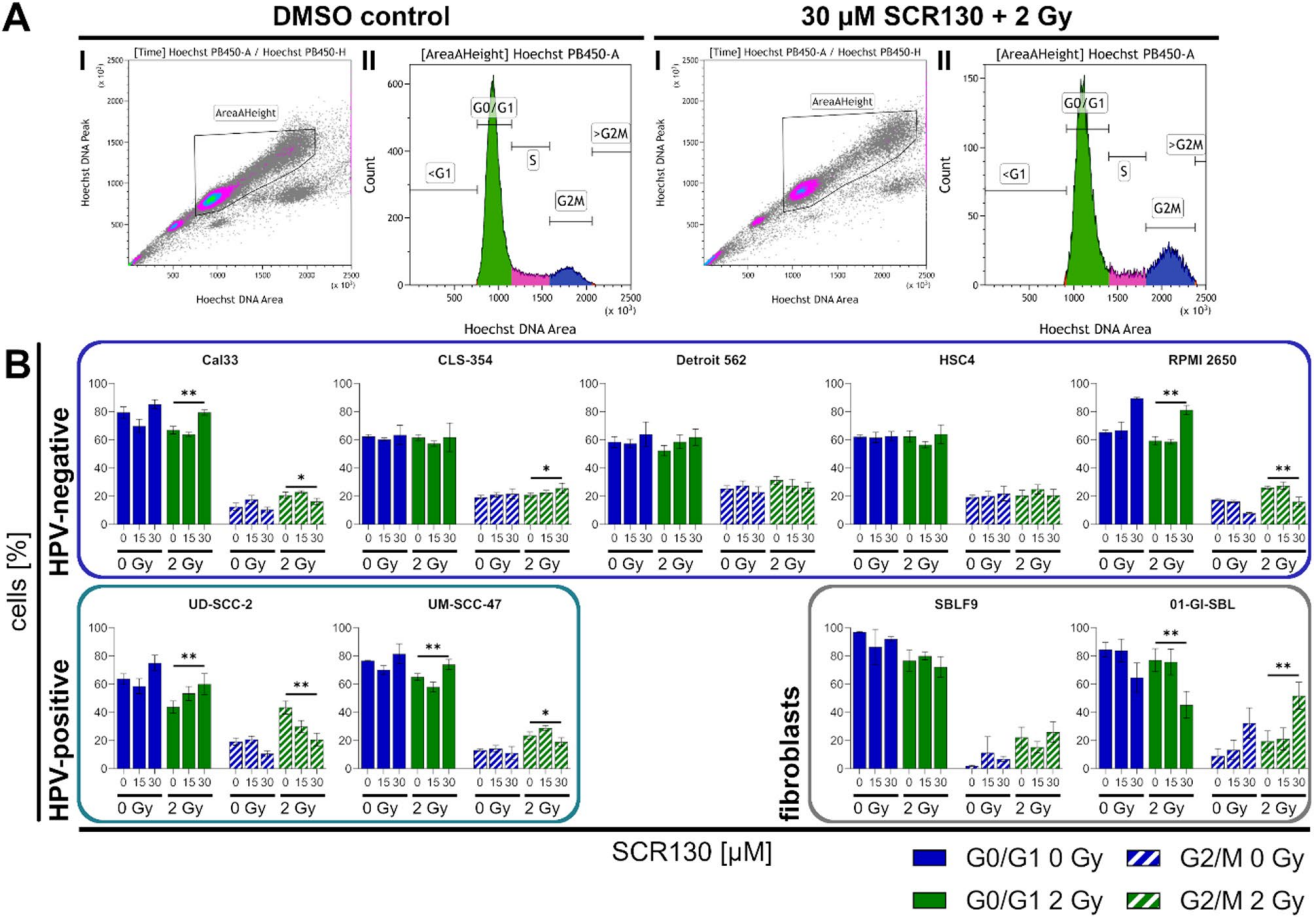
Flow cytometry analysis of cell cycle distribution. (**A**) Gating strategy: Exemplary flow cytometry results of UM-SCC-47 treated with vehicle control (DMSO) or combined treatment (30 µM SCR130 + 2 Gy). At first, doublets and cell debris (I) were excluded. Afterwards, the amount of cells in the different cell cycle phases was determined according to their DNA content (II). (**B**) Cell cycle analysis: Cells in G0/G1 phase (filled) and G2/M phase (dashed) divided in unirradiated (0 Gy, blue) and irradiated (2 Gy, green) samples in different HPV-negative and HPV-positive HNSCC cell lines and healthy fibroblasts after treatment. Cells were treated with 15 or 30 µM SCR130; half of the samples additionally received 2 Gy IR 3 h afterwards. Vehicle control was treated with the same volume DMSO as used for 30 µM SCR130 (0 Gy). Statistically significant differences were calculated between 2 Gy and 30 µM SCR130 + 2 Gy (for G0/G1 and G2/M) by two-tailed Mann-Whitney test to evaluate the radiosensitizing effect of SCR130. Each value represents mean ± SD (*n* ≥ 4). *p = 0.028, **p = 0.004, except UD-SCC-2 G0/G1 **p = 0.008.

Hoechst staining of the DNA allowed the determination of the cell cycle phase after tumor cell treatment. At first, doublets and cell debris were excluded. Secondly, the cell fractions were differentiated depending on their bound Hoechst dye which corresponds with their DNA content varying in the different cell cycle phases (Fig. 3A).

In all HNSCC cell lines treatment with 30 µM SCR130 alone slightly induced G0/G1 block compared to DMSO control but contrary in fibroblasts. A significant increase (*p* = 0.004–0.008, two-tailed Mann-Whitney test, 2 Gy vs. 30 µM SCR130 + 2 Gy) of cells in G0/G1 phase by the combined treatment compared to IR alone was shown for Cal33, RPMI 2650, UD-SCC-2, and UM-SCC-47. In contrast, the number of cells in G0/G1 phase decreased significantly in 01-GI-SBL (*p* = 0.004). Corresponding, the amount of cells in G2/M phase after the combined treatment decreased significantly (*p* = 0.004–0.028) in Cal33, RPMI 2650, UD-SCC-2, and UM-SCC-47; the only HNSCC cell line where the amount of cells in G2/M phase increased was CLS-354 as well as in 01-GI-SBL. The response of the single cell lines to the treatment did not correlate with their HPV-status but was cell line specific (Fig. 3B).

### SCR130 barley reduces colony formation

**Fig. 4:**
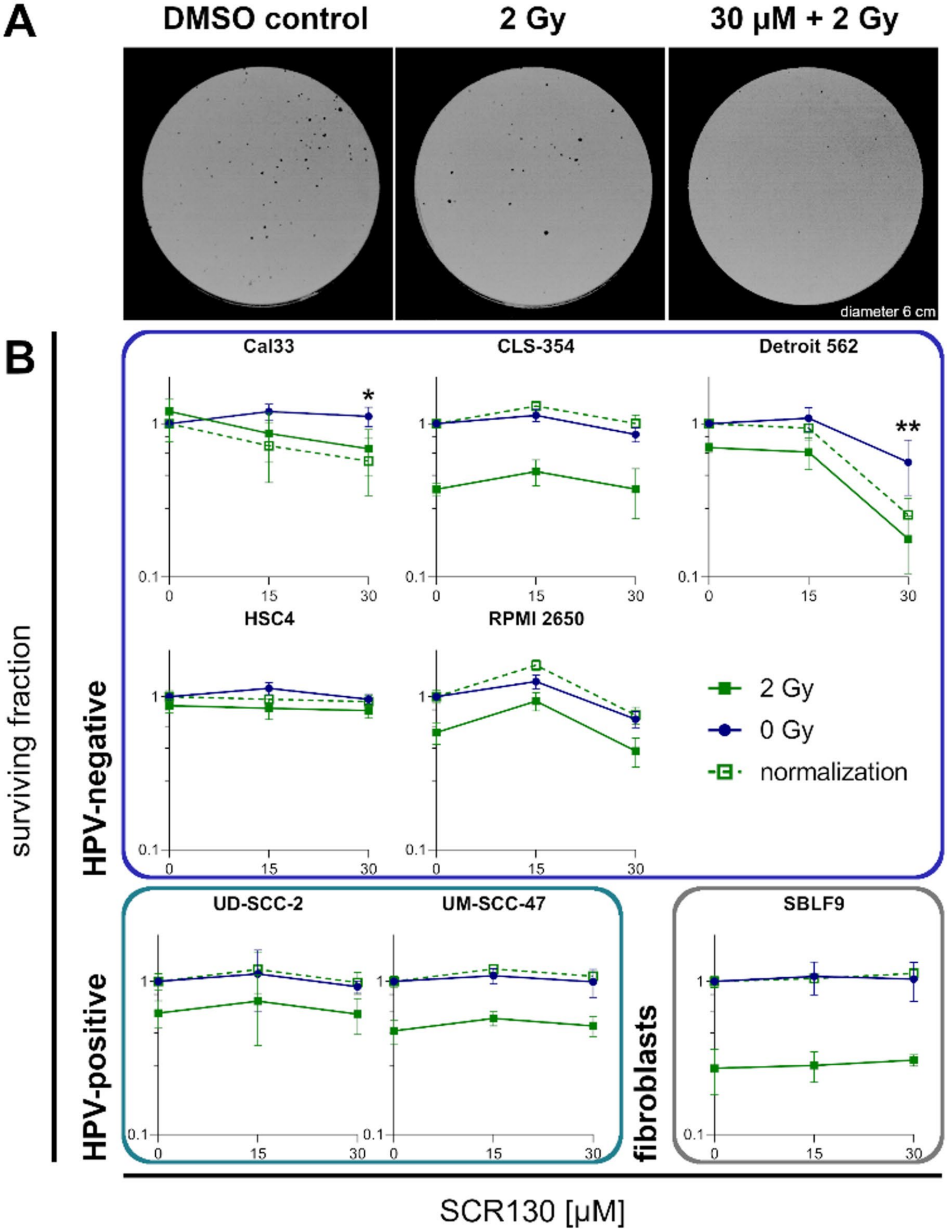
Colony formation assay. (**A**) Representative results of the colony formation assay: stained Petri dishes with Detroit 562 after 10 days of incubation. Cells were treated with vehicle control (DMSO), 2 Gy of IR, or combined treatment (30 µM SCR130 + 2 Gy). (**B**) Surviving fraction (SF) of different HPV-negative and HPV-positive HNSCC cell lines and healthy fibroblasts after treatment. Cells were treated with 15 or 30 µM SCR130; half of the samples additionally received 2 Gy IR 3 h afterwards. Vehicle controls were treated with the same volume DMSO as used for 15 and 30 µM SCR130 (0 Gy) respectively and were used for calculating the SF. 2 Gy data were normalized to the vehicle control that received the same volume DMSO as used for 30 µM SCR130. Statistically significant differences were calculated between 2 Gy and 30 µM SCR130 + 2 Gy by one-tailed Mann-Whitney test to evaluate the radiosensitizing effect of SCR130. Each value represents mean ± SD (*n* ≥ 4). *p = 0.029, **p = 0.001.

With the colony formation assay we were able to determine the ability of the cells to form colonies after treatment (Fig. 4A). In general, the radiation sensitivity of the cell lines varied from SBLF9 and CLS-354, where IR alone strongly reduced the SF, to HSC4, which barely responded to IR. Because IR alone already reduced the SF, making it difficult to estimate the additional effect of SCR130, we normalized the irradiated samples to the DMSO control. To do so we shifted the 2-Gy-line parallel into the starting point of the 0-Gy-line (SF = 1). This resulted in the same starting point for the 0-Gy- and 2-Gy-lines in the diagram, which makes it easier to understand the influence of SCR130 with or without IR. The combined treatment resulted in a significant decrease of the SF and therefore in the favored radiosensitization depending on SCR130 concentration in Cal33 and Detroit 562 whereas the other cell lines only reacted to the IR but not to additional SCR130 treatment. 01-GI-SBL is a primary fibroblastic cell line that did not grow properly with the small cell density necessary for colony formation assay. Therefore, they did not form any colonies and no data are available for this cell line (Fig. 4B).

### SCR130 does not influence the elimination of IR-induced γH2AX foci

**Fig. 5:**
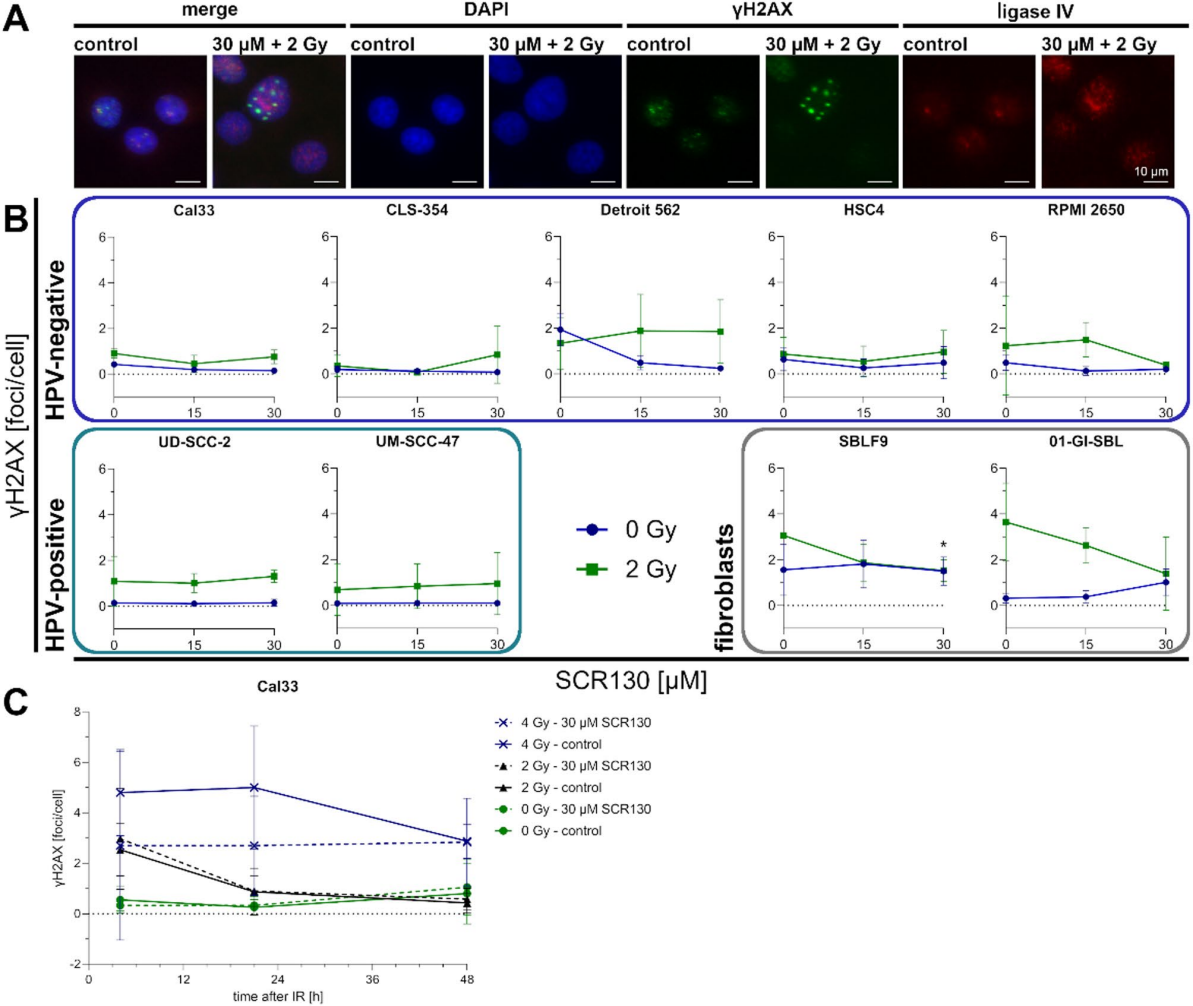
Immunostaining. (**A**) Representative images of immunostained Cal33. Untreated cells (control) and combined treatment (30 µM SCR130 + 2 Gy) are compared. DAPI (blue), γH2AX (488 nm, green), and ligase IV (555 nm, red) are shown separately. DAPI, γH2AX, and ligase IV are merged. Scale bar represents 10 μm. (**B**) Quantification of γH2AX foci/cell in different HPV-negative and HPV-positive HNSCC cell lines and healthy fibroblasts after 24 h of inhibitor treatment. Cells were treated with 15 or 30 µM SCR130; half of the samples additionally received 2 Gy IR 3 h afterwards. Fixation was done 21 h after irradiation. Controls were completely untreated. Statistically significant differences were calculated between 2 Gy and 30 µM SCR130 + 2 Gy by two-tailed Mann-Whitney test to evaluate the radiosensitizing effect of SCR130. Each value represents mean ± SD (*n* = 4; except Detroit 562 15 µM/0 Gy *n* = 4; Detroit 562 30 µM/0 Gy, UD-SCC-2 30 µM/2Gy, 01-GI-SBL 0 µM/0 Gy, 0 µM/2 Gy, 15 µM/2 Gy *n* = 3). *p = 0.029. (C) Number of γH2AX foci/cell 4, 21, and 48 h after irradiation with 2–4 Gy IR with or without 30 µM SCR130 in Cal33. Each value represents mean ± SD (*n* ≥ 3).

Cells were treated and stained with target specific antibodies. Ligase IV signal was detectable in all tested cell lines (data not shown) (Fig. 5A).

Remaining DNA damage after 24 h of treatment was quantified counting γH2AX foci per nucleus. In all cell lines there were nearly no foci in the untreated control. Except for Detroit 562 (1.9 foci/cell) and SBLF9 (1.6 foci/cell) there were less than 1 foci/cell. The remaining foci level was elevated by IR up to 3.6 foci/cell in 01-GI-SBL. SCR130 did not influence the clearance of γH2AX foci after 24 h except in RPMI 2650, SBLF9, and 01-GI-SBL where the combined treatment reduced the foci number slightly compared to IR alone (Fig. 5B).

To detect changes in the dynamics of DNA repair, γH2AX level was determined 4, 21, and 48 h after irradiation exemplary in Cal33. Moreover, a higher dose of 4 Gy IR was applied additionally. As expected, the initial damage was proportional to the applied dose and decreased from 4 to 48 h after irradiation. The treatment with SCR130 neither influenced the number of γH2AX foci/cell nor their clearance (Fig. 5C).

### SCR130 combined with IR regulates mRNA expression of ligase IV

mRNA expression of several cell cycle regulation proteins and DNA repair proteins was analyzed with qRT-PCR. The relative expression of treated samples compared to DMSO control was calculated.

**Fig. 6:**
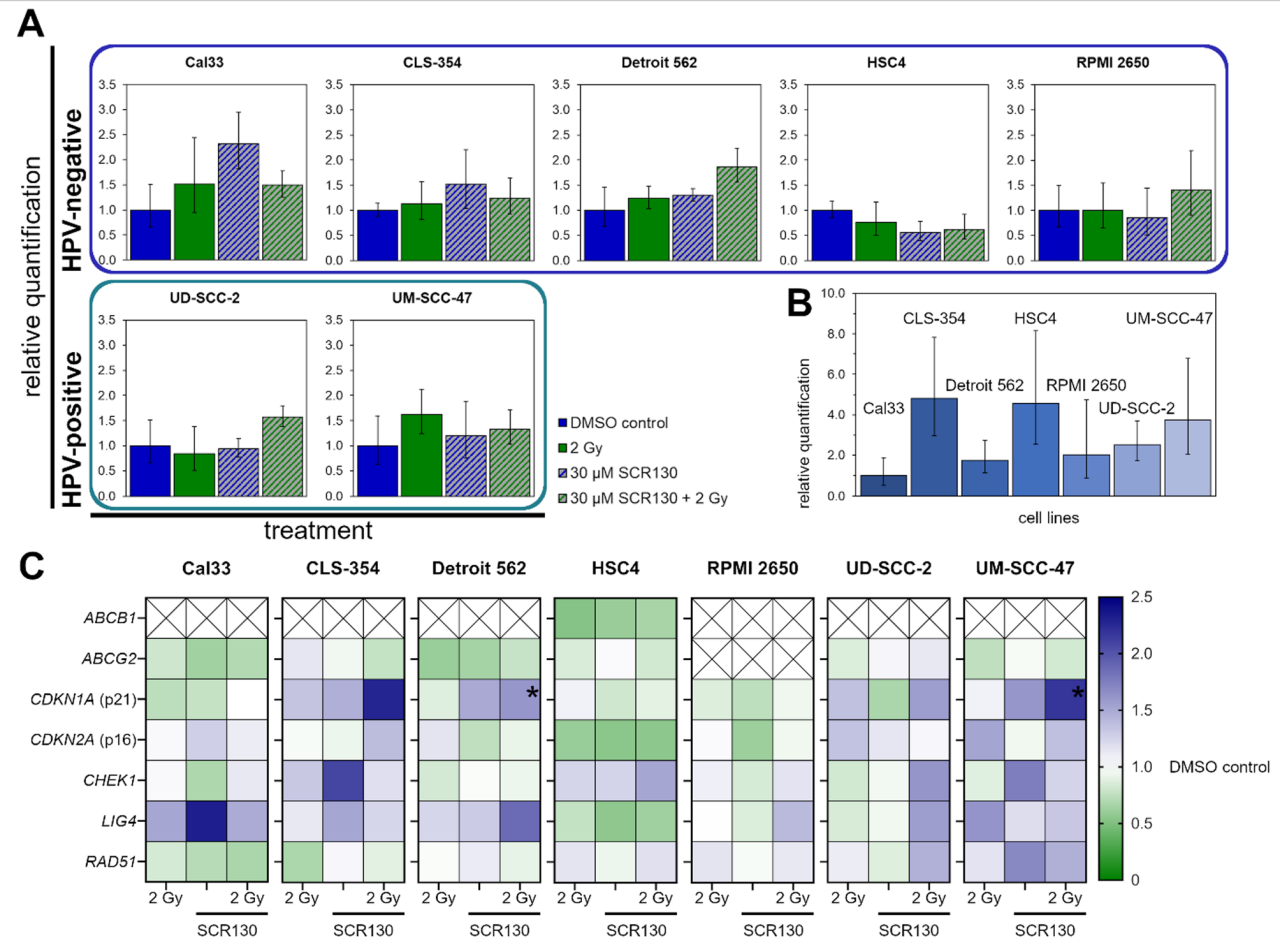
qRT-PCR for mRNA expression analysis. (**A**) Relative quantification of *LIG4* mRNA compared to DMSO control in different HPV-negative and HPV-positive HNSCC cell lines after treatment. Cells were treated with 30 µM SCR130; half of the samples additionally received 2 Gy IR 3 h afterwards. Vehicle control was treated with the same volume DMSO as used for 30 µM SCR130 (0 Gy). (**B**) *LIG4* baseline expression in the different cell lines: relative quantification of DMSO controls compared to Cal33. (C) Regulation of different cell cycle control and DNA repair genes in the different cell lines after treatment (according to A, from left to right: 2 Gy, 30 µM SCR130, 30 µM SCR130 + 2 Gy) relative to DMSO control (white). Upregulation is represented by increasing intensity of blue color, downregulation by green color. Boxes with “x” (*ABCB1*, *ABCG2*) represent undetectable low expression. Statistically significant differences were calculated between 2 Gy and 30 µM SCR130 + 2 Gy (log transformed data of relative gene expression) by one-tailed Mann-Whitney test to evaluate the radiosensitizing effect of SCR130. Each value represents mean ± SD (*n* = 4), applies for **A**, **B**, and **C**. *p = 0.014.

The target of SCR130, ligase IV, was slightly upregulated by the combined treatment in all HNSCC cell lines except HSC4 but only in Cal33, CLS-354, Detroit 562, and UM-SCC-47 by IR or SCR130 alone. HSC4 was exceptional because the ligase IV level decreased in all treatments (Fig. 6A).

Moreover, we evaluated the baseline expression of *LIG4* in cells only treated with DMSO. Cal33 had the lowest level of *LIG4* mRNA expression whereas CLS-354 and HSC4 had higher levels of *LIG4*. Overall, the amount of expressed *LIG4* mRNA depended on the cell line (Fig. 6B).

To better understand cell line specific escape mechanisms, the mRNA expression of the two cellular export proteins ABCB1 and ABCG2, which are known for causing treatment resistance^31^, was quantified. *ABCB1* was only detectable for HSC4 where it was downregulated trough IR, SCR130, and the combination. In the other cell lines the amount of mRNA for this gene was too low for detection with the used qRT-PCR protocol. *ABCG2* was downregulated by all treatment options in all cell lines (RPMI 2650 not detectable). CDKN1A, CDKN2A, and CHEK1 are essential proteins for cell cycle control^32–34^; therefore, their mRNA expression was quantified to explain SCR130’s influence on cell cycle distribution. *CDKN1A* was strongly upregulated by the combined treatment in CLS-354, Detroit 562, UD-SCC-2, and UM-SCC-47. As *CDKN1A* is associated with senescence^35^, we additionally measured C12FDG signal as a senescence marker exemplary in Cal33 and UM-SCC-47. While in Cal33 the amount of C12FDG-positive cells was not increase by IR, SCR130, or the combined treatment, in UM-SCC-47 C12FDG-positivity was induced by IR but not by SCR130 (compare to Supplementary Fig. 1). In contrast to *CDKN1A*, *CDKN2A* was only upregulated in CLS-3554 and UM-SCC-47. *CHEK1* was upregulated by SCR130 in CLS-354 and UM-SCC-47 and downregulated again by IR. In HSC4 and UD-SCC-2 the combined treatment resulted in an upregulation of *CHEK1* whereas its expression was barely influenced in the other cell lines and conditions. To elucidate the individual DDR of the HNSCC cell lines in more detail, we quantified mRNA expression of *RAD51* because of its role in HR^36^. *RAD51* was nearly constant in the different treatment options. Only in Cal33 there was a downregulation in all treatments and in CLS-354 after 2 Gy IR. Whereas *RAD51* was upregulated in UD-SCC-2 after 2 Gy IR and in UM-SCC-47 because of SCR130 and the combined treatment (Fig. 6C).

### Formulation and time-dependent optimization of SCR130 treatment

**Fig. 7:**
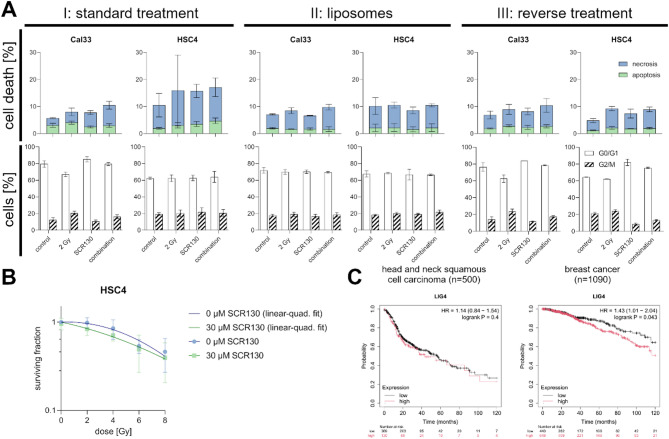
Optimization of SCR130 treatment. (**A**) Comparison of cell death and cell cycle distribution resulting from (I) standard treatment (see Figs. 2, 3, 30 µM SCR130) with two different alternative treatment schemes in Cal33 and HSC4. (II) Treatment with liposomes: liposomes were loaded with 10 mM SCR130 solution and diluted according to the standard treatment 1:333. Each value represents mean ± SD (*n* = 2). (III) Reverse treatment: 30 µM SCR130 were added 3 h after application of 2 Gy IR instead before. Each value represents mean ± SD (*n* = 2). All the other steps were carried out according to the standard treatment. Control: DMSO according to 30 µM SCR130 (I and III), unloaded liposomes diluted 1:333 in the treated volume (II); 2 Gy: 2 Gy IR; SCR130: 30 µM SCR130 (I and III), 1:333 dilution of liposomes loaded with 10 mM SCR130 in the treated volume (II); combination: combination of SCR130 and 2 Gy IR according to the treatment scheme. (**B**) Colony formation assay: Treatment of HSC4 with 30 µM SCR130 and increasing single doses of IR up to 8 Gy (instead of 2 Gy) according to the standard treatment. The control was treated with the same volume DMSO as used 30 µM SCR130 and was used for calculating the SF. Normalized data to DMSO control and linear quadratic fit of 0 µM and 30 µM SCR130 are shown. Each value represents mean ± SD (*n* = 4). (C) Overall survival of patients with HNSCC and breast cancer depending on their ligase IV expression (mRNA data from RNA seq). Data and analysis from publicly available Kaplan-Meier Plotter database^37^.

We compared the standard treatment with two other treatment schemes for optimizing SCR130’s effect in Cal33 and HSC4. Especially HSC4 did not react to SCR130 treatment in any of the previous assays, which makes it a suitable candidate for optimization. Therefore, we delivered SCR130 in liposomes to facilitate its uptake into the cells, changed the order of inhibitor addition and irradiation, and increased the dose of IR. The results for the standard treatment are the same as presented in Figs. 2 and 3. Neither cell death nor cell cycle distribution were influenced by the delivery of SCR130 in liposomes or the reverse application of IR followed by SCR130 (Fig. 7A).

To avoid the influence of individual radioresistance of HSC4, which could make higher doses necessary for this cell line compared to the other cell lines to measure SCR130’s effect, the dose of IR was escalated from 2 Gy to 8 Gy. The SF decreased with increasing dose application but the addition of SCR130 could not enhance the radiation effect (Fig. 7B).

Kaplan-Meier Plotter is a publicly available tool summarizing gene expression data from several genome databases and published clinical survival data. It enables calculating Kaplan-Meier plots discriminating between patients with high or low expression of a gene of interest, in our case *LIG4*, to validate predictive biomarkers^37^. Using Kaplan-Meier Plotter we investigated the influence of ligase IV expression on the overall survival of HNSCC and breast cancer patients. In fact, the tendencies differ for those entities. While there is no impact for HNSCC patients, low ligase IV expression is beneficial for breast cancer patients (Fig. 7C).

## Discussion

The efficacy of RT is strongly dependent on the capability of tumor cells to repair IR-induced DNA DSBs^23^. Therefore, inhibition of DNA DSB repair using SMIs is a promising approach to increase radiation sensitivity^23^. According to Kumari et al., mutations in the ligase IV encoding gene of a cervical cancer cell line sensitize the cells to IR compared to their wild type^21^. The SMI SCR130 presented by Ray et al. allows blocking of NHEJ by inhibiting ligase IV and could therefore radiosensitize cancer cells^27^.

We treated a panel of seven HNSCC and two healthy fibroblast cell lines with SCR130 combined with IR to investigate its potential for RT of HNSCC cancer patients. Even if the ability of SCR130 to inhibit NHEJ was described in a previous study^27^, its radiosensitizing effect was low and highly cell line specific. We found that SCR130s’ effect was limited to an increased G0/G1 phase and increased p21 expression, while the influence on cell death, clonogenicity, and DNA damage was minimal. To explain the low effectiveness, we analyzed the regulation of several DDR genes and suggest the upregulation of Ligase IV as a possible escape mechanism. Even adjustments of the experimental setup did not lead to improved radiosensitization by SCR130.

### SCR130 combined with IR induces cell death heterogeneously

There was a significant increase of IR-induced cell death after the combined treatment in five of seven HNSCC cell lines (compare to Fig. 2). In contrast, two of them did not respond at all exactly as the healthy fibroblasts. The resistance of the healthy cells against the treatment is beneficial because it implies that the treatment would be less hazardous for healthy than for cancerous tissue. The HPV-positive cell lines UD-SCC-2 and UM-SCC-47 were more sensitive to the combined treatment and died more effectively, while the HPV-negative ones were heterogeneously sensitive to SCR130. It is already well described that HPV-induced HNSCCs are more sensitive towards therapy and have a better clinical outcome than HPV-negative ones^6–8^. Nevertheless, except in HSC4 and RPMI 2650 in all of the HNSCC cell lines an increased dead population was detectable after combined treatment compared to IR alone, which indicates a radiosensitizing effect of SCR130 in some HNSCC cell lines. These findings represent the heterogeneity of HNSCC patients regarding molecular characteristics, treatment response, and clinical outcome^38^. Mutations in proteins of signaling pathways, cell death, and cell cycle control are - among various others - frequently altered; yet, no distinct molecular patterns or biomarkers are validated allowing classification of patients^38^. HNSCCs’ heterogeneity could be explained by the different origin in the head and neck region, diverse molecular profiles, and - of course - HPV-status^39^.

### Antiproliferative potential of SCR130 resulting in G0/G1 arrest

Since radiation sensitivity and therefore the response to RT varies depending on the cell cycle phase of cells^40^, it is essential to understand the effect of the treatment on cell cycle distribution. Already in the 1960s, Sinclair concluded that the radiation sensitivity of cells increases from S phase over G0/G1 phase to G2/M phase^40^. Problematically, the only HNSCC cell line replying to the combined treatment with an increased number of cells in G2/M phase compared to IR alone was CLS-354. Equally, the healthy cell line 01-GI-SBL, which should be spared from radiosensitization ideally, showed a G2/M block. IR alone induced a G2/M block in all tested cell lines as already shown by Liu et al.^41^. Interestingly, this was compensated by the ligase IV inhibitor SCR130 in Cal33, RPMI 2650, UD-SCC-2, UM-SCC-47, and slightly in Detroit 562, as evidenced by a reduction of cells in G2/M phase and corresponding increase in cells in G0/G1 phase after combined treatment compared to IR alone. Remarkably, it is well described for tumor cells in contrast to healthy tissue that the regulation of progression from G1 to S phase is commonly impaired and tumor cells does not arrest in G0/G1 phase but proceed in the cell cycle to the S phase^42^. Consequently, we would have not expected a G0/G1 arrest in the HNSCC cell lines. Wilson et al. showed that cell cycle arrest in the G1 phase in a breast cancer cell line correlates with induction of senescence, but we could not detect senescence after treatment by C12FDG staining^43^. Preferably, the induction of G0/G1 arrest can be anti-proliferative^44^ and, according to Foy et al., G1 arrest can result in uncontrolled growth, which is toxic for the cells^45^.

Even if there was no radiosensitizing effect by arresting cells to G2/M phase, which would be preferable for fractionated treatment scheme^46^, reduced proliferation and toxic cell growth in G1 arrest can compensate for this. The cell cycle distribution of HSC4 and SBLF9 fibroblasts was not modified by the combined treatment of SCR130 and IR. As already seen and discussed in cell death analysis, HPV-positive cell lines were arrested by the combined treatment in G0/G1 whereas HPV-negative ones responded heterogeneously.

### Limited effect of SCR130 and IR on clonogenicity of HNSCC cell lines

In contrast to cell death, clonogenicity was clearly limited by the combined treatment only in Cal33 and Detroit 562. Especially for Cal33 the detected effect was much larger in the colony formation assay than in cell death analysis. Preferably, SBLF9 fibroblasts were in general very radiation sensitive compared to the HNSCC cell lines but the additional SCR130 treatment did not reduce the SF in these cells. Nevertheless, the influence of SCR130 on clonogenicity is limited and highly cell line specific.

### SCR130 does not inhibit elimination of IR-induced DNA damage

We used the sensor protein γH2AX for DNA DSBs^47^ to identify remaining DNA damage after 24 h repair time. As expected, DNA damage was higher in irradiated samples but there was no reduced clearance of γH2AX foci resulting from SCR130 treatment in the HNSCC cell lines. The dynamic of DNA damage was not influenced by SCR130. The fibroblast cell lines SBLF9 and 01-GI-SBL showed more DNA damage than the HNSCC cell lines after IR but in contrast to them a stronger decrease after treatment with SCR130. The high radiation sensitivity of SBLF9 was also detectable in colony formation assay. We conclude that the fibroblasts’ repair capacity is increased by SCR130 in contrast to HNSCC cell lines. This would be preferable for SCR130 treatment of cancer tissue while sparing healthy cells. The γH2AX foci after IR in HPV-positive cell lines compared to HPV-negative ones showed higher radiation sensitivity of HPV-positive cell lines.

### SCR130 combined with IR regulates DDR proteins

The functional assays revealed a heterogenous outcome of the different cell lines resulting from the combined treatment of SCR130 and IR. Especially HSC4 and RPMI 2650 could handle the treatment very well. Therefore, we measured the mRNA expression of several genes involved in DDR to explain the different outcomes and elucidate possible escape mechanisms.

*ABCB1* and *ABCG2* are genes encoding for ATP binding cassette proteins associated with multidrug resistance^31^. *ABCB1* encodes for the P-glycoprotein that contributes to drug resistance by avoiding drugs from entering cells in an expression dependent manner^48^.

It is known that its overexpression on cancer cells leads to multidrug-resistance by actively transporting therapeutics outside the cell^49^. Same is true for *ABCG2*^31^. In general, the expression of both genes was very low and undetectable in some cell lines. For better understanding a pre-amplification before qRT-PCR could be an option. Nevertheless, there was a downregulation of *ABCB1* and *ABCG2* in all detectable conditions and cell lines. Therefore, we rejected the hypothesis that an upregulation of efflux proteins may be an escape mechanism of the cell lines for the SCR130 treatment.

To better understand the effect of SCR130 on cell cycle distribution, we measured the expression of *CDKN1A*, *CDKN2A*, and *CHEK1*. *CDKN1A* encodes for cyclin-dependent kinase inhibitor 1 also known as p21; it is relevant for cell cycle arrest in all cell cycle phases, which is necessary in several physiological processes and DDR to ensure genomic stability^32^. Perucca et al. showed that missing of p21 could lead to an uncontrolled entry into S-phase and therefore to proliferation instead of cell cycle arrest^50^. Mauro et al. additionally described that p21 deficient cells are sensitive to DNA damage inducing agents and use NHEJ for DNA damage repair concluding that p21 reduces use of NHEJ and increases HR^51^. Comparing the regulation of *CDKN1A* with the results from cell cycle analysis revealed that Cal33, Detroit 562, UD-SCC-2, and UM-SCC-47 showed G0/G1 arrest in the combined treatment compared to IR corresponding with an increased expression of *CDKN1A*. Foy et al. also showed that an elevated p21 level in tumor cells correlates with G1 arrest^45^. In glioblastoma cell lines Mansour et al. demonstrated that increasing amount of p21 is associated with radiation-induced senescence and reduced proliferation^35^. Nevertheless, considering C12FDG signal, there was no indication that SCR130 induces senescence. The only cell line with a G0/G1 arrest but without upregulation of *CDKN1A* was RPMI 2650. *CDKN1A* upregulation could be an explanation for the G0/G1 block that was induced by SCR130 and an indicator for preferring HR instead of NHEJ^51^, which could explain why remaining DNA damages in γH2AX assay were unaffected by SCR130 treatment. For the non-responder cell lines HSC4 and RPMI 2650 was no upregulation detectable.

Another important tumor suppressor gene is *CDKN2A* encoding p16; its activity restricts the transition of cells from G1 to S phase by regulating the retinoblastoma protein pathway^33^. Impaired function of p16 is very common in cancer so it remains unclear if the used cell lines are able to regulate p16 expression or build a functional protein^33^. Cal33, UD-SCC-2, and UM-SCC-47 upregulated *CDKN2A* because of the combined treatment which can explain their G0/G1 arrest whereas *CDKN2A* was also upregulated in CLS-354 but no G0/G1 block occurred. By downregulation of *CDKN2A* Detroit 562, HSC4, and RPMI 2650 could try to avoid treatment-induced cell cycle arrest for DNA damage repair.

If DNA damage occurs the intra-S and G2/M checkpoints are activated by cell cycle control protein CHEK1 and ATR causing cell cycle arrest^34^. Therefore, reduced CHEK1 activity can induce cell death^34^. *CHEK1* upregulation was detectable after combined treatment in all cell lines except Detroit 562 but only resulting in G2/M block in CLS-354. Probably the effect of CDKN1A and CDKN2A dominates or CHEK1 functionality is impaired. Wang et al. showed that in a “stem cell-like population of nasopharyngeal carcinoma cells” upregulation of *CHEK1* ended in radioresistance^52^. In HSC4 and UD-SCC-2, which did not react at all or did not react in the colony formation assay respectively, the upregulation of *CHEK1* after combined treatment was remarkable and could be a possible escape mechanism from SCR130 in those cell lines.

In six out of seven HNSCC cell lines - all except HSC4 - upregulation of *LIG4* was detectable after combined treatment whereas it was only upregulated in four cell lines after SCR130 alone. Srivastava et al. showed for the ligase IV inhibitor SCR7 that cell lines with high amount of ligase IV could escape inhibitor treatment^25^. This leads to the conclusion that all the cell lines – except HSC4, which probably have other cell line specific escape mechanisms available - tried to overcome the inhibition to deal with the IR-induced DNA damage. The upregulation of *LIG4* after treatment limits the potential of SCR130 as a radiosensitizer. Notably, the cell lines differed in their *LIG4* baseline level but there was no correlation between the baseline level and response to the treatment.

As a marker of HR, we checked the expression of *RAD51*. As HR relies on a homologous DNA repair and strand invasion, RAD51 is relevant in these steps of DNA damage repair via HR^36^. An upregulation of *RAD51* was shown to be a predictive marker for chemoresistance and poor prognosis *in vivo* and *in vitro*^53,54^. *RAD51* was barely regulated by our treatment on the mRNA level; only in the HPV-positive cell lines UD-SCC-2 and UM-SCC-47 there was an upregulation after combined treatment and a downregulation in Cal33. Comparable to these findings Holcomb et al. demonstrated that the expression of DNA repair proteins in HNSCCs correlates with the HPV-status; they found a higher expression in HPV-positive HNSCCs, especially an increase of *RAD51*^55^.

### Optimized treatment protocol does not increase the effect of SCR130

As shown in the functional assays, SCR130 has the potential to increase radiation sensitivity by ligase IV inhibition. Nevertheless, some cell lines only slightly responded to the treatment or could escape completely. Some possible escape mechanisms were already discussed. To increase the effect of SCR130, two optimized treatment schemes were used. Due to possible low cell membrane permeability, we loaded liposomes with SCR130 to improve drug uptake into the cells. We chose liposomes as a promising drug delivery system in our study because they are biocompatible, non-toxic themselves, can enhance solubility of drugs, and can optimize cellular drug uptake due to their cell membrane-like lipid structure^30,56^. Nevertheless, there was no increased cell death or changed cell cycle distribution resulting from the liposome treatment compared to the standard treatment. This leads us to the conclusion that SCR130 uptake of the cells or its solubility is not the limiting factor. Our group established the standard treatment scheme with addition of SMI 3 h prior to IR in several studies^57–59^. Nevertheless, there is evidence that SMI probably need IR-induced damage before their addition to function, as shown for AT406 known as Xevinapant^60^. We could show that the reverse treatment scheme did not increase SCR130s’ efficacy. Also, we checked if HSC4, which showed up as non-responder, may have an individual radiation sensitivity that is lower than for the other cell lines. But even with higher doses of IR there was no increased effect of SCR130. As we rejected low cellular uptake, treatment procedure, and individual radiation sensitivity as reasons for SCR130’s limited efficacy, it is likely that it is structure-dependent or the cells can handle the treatment because of intrinsic escape mechanisms.

Another option is that SCR130 is not best suitable for treatment of HNSCC but another tumor entity. Analysis of entity-specific Kaplan-Meier plots revealed that there is no influence of ligase IV expression on the overall survival of HNSCC patients but for breast cancer patients, where a low ligase IV expression in the cancer tissue was favorable for overall survival^37^. Overall, the influence of ligase IV expression on survival is heterogenous and entity-specific^37^. Grupp et al. for example also showed that poor clinical prognosis in prostate cancer correlated with ligase IV expression^61^. Therefore, an analysis of SCR130 in another entity could be promising.

To sum it up, we showed that the combination of IR with SCR130 has the potential to radiosensitize HNSCC while sparing healthy fibroblast cell lines. Even if SMI are administered systematically, there are a few characteristics of normal tissue that enable targeting cancer cells and sparing healthy ones. Tumor cells proliferate more than healthy cells and therefore accumulate more replication-induced damage, which will persist because of inhibited DNA repair^62^. Moreover, tumor cells are known to have impaired DNA repair while healthy cells have several repair pathways available^62^. Especially for HR deficient cells^17–19^ targeting NHEJ is promising because of the important role of those pathways in DNA DSB repair^13^. Srivastava et al. already showed that HR deficiency sensitizes cell lines to ligase IV inhibition^25^. The HPV-positive cell lines UD-SCC-2 and UM-SCC-47, which already have a better prognosis^6–8^, reacted with increased cell death and G0/G1 arrest. The reaction of the HPVnegative cell lines was more heterogenous only leading to increased cell death in one of five cell lines, reduced SF and G0/G1 arrest in two of five cell lines, and G2/M block in one of five cell lines. Unfavorable results in HPV-negative cell lines are presumably caused by cell line origin and genetic differences, for example in cell death and cell cycle regulation, which is also true for clinical outcome in patients^38,39^.

Considering the vital role^20^ of ligase IV in NHEJ, this low efficacy regarding cell death, colony formation assay, and DNA damage is noteworthy. Nevertheless, we suggest that SCR130 has antiproliferative effects seen by increased G0/G1 fraction. Moreover, we elucidated some possible escape mechanisms importantly the upregulation of *LIG4* and could exclude others like efflux proteins or drug uptake and solubility. Especially HSC4 and RPMI 2650 effectively escaped the treatment without knowing why yet. A possible escape mechanism that need to be checked further could be the upregulation of alternative repair mechanisms because there is broad evidence that NHEJ deficient cells can switch to alternative end-joining for DNA repair^63,64^. Another possibility would be that cells already have mutations in their ligase IV encoding gene or alternative genes of radiation response, therefore SCR130 could not work^10^.

Our study investigated the radiosensitizing effect of the ligase IV inhibitor SCR130 on HNSCC cell lines. Even if there were slight effects detectable, they were weak and strongly cell line specific. Yet, there is no final explanation if those limited effects are caused by intrinsic escape mechanisms of the cell lines or the lack of efficacy of the drug. Future research should focus on the cell line specific differences in the DDR. Moreover, it remains unclear if the low efficacy of SCR130 is caused by its chemical structure and that the SMI therefore is insufficient itself. SCR130 is already an improved structure of SCR7 and meanwhile there are several modifications available that may probably improve the performance of the ligase IV inhibition^27,65^.

## Conclusions

We characterized the radiosensitizing effect of the ligase IV inhibitor SCR130 on HNSCC and healthy fibroblast cell lines. Radiosensitization regarding cell death was possible but cell line specific, while it was limited in colony formation assay. Combined treatment resulted in G0/G1 arrest, which was accompanied by upregulation of *CDKN1A* (p21) and *LIG4*. Therefore, we suggest that SCR130 has anti-proliferative potential but there are also non-responder cell lines. Considering the vital role of ligase IV in DNA DSB repair, the limited effects of SCR130 in combination with IR are unexpected and the heterogenous reaction of the different cell lines is noteworthy. We elucidated the upregulation of *LIG4* after SCR130 treatment as a possible escape mechanism. Overall, our study indicates that SCR130 is not able to inhibit NHEJ in HNSCC cell lines and therefore is not a sufficient radiosensitizer.

## Electronic supplementary material

Below is the link to the electronic supplementary material.

Supplementary Material 1

## Data Availability

All data generated and presented during this study are available from the corresponding author on reasonable request. Publicly available data presented in this study can be accessed on the following website: KMplot (https://kmplot.com/analysis/, accessed on September 09, 2024).

## Abbreviations

ABCB1: ATP binding cassette subfamily B member 1
ABCG2: ATP binding cassette subfamily G member 2
ACTB: Actin beta
APC: Allophycocyanin
ATM: Ataxia telangiectasia mutated
ATR: Ataxia telangiectasia and Rad3-related protein
BSA: Bovine serum albumin
Cardiolipin: 18:1 1’,3’-bis[1,2-dioleoyl-sn-glycero-3-phospho]-glycerol
CDKN1A: Cyclin dependent kinase inhibitor 1 A
CDKN2A: Cyclin dependent kinase inhibitor 2 A
cDNA: Complementary DNA
CHEK1: Checkpoint kinase 1
C12FDG: 5-Dodecanoylaminofluorescein Di-β-D-Galactopyranosid
DAPI: 2-(4-Amidinophenyl)-1 H-indole-6-carboxamidine
DMSO: Dimethyl sulfoxide
DDR: DNA damage response
DNA-PKcs: DNA-dependent protein kinase catalytic subunit
DOPG: 18:1 1,2-dioleoyl-sn-glycero-3-phospho-(1’-rac-glycerol)
DPBS: Dulbecco’s phosphate buffered saline
DSB: Double strand break
EDTA: Ethylenediaminetetraacetic acid
FBS: Fetal bovine serum
gDNA: Genomic DNA
Gy: Gray
HMBS: Hydroxymethylbilane synthase
HNSCC: Head and neck squamous cell carcinoma
HPV: Human papilloma virus
HR: Homologous recombination
IR: Ionizing radiation
Ku70/Ku80: Dimeric Ku70/Ku80 protein complex
LIG4: DNA ligase 4
mTOR: Mechanistic target of rapamycin kinase
NHEJ: Non-homologous end joining
NTA: Nanoparticle tracking analyzer
NTC: No template control
PAXX: Paralog of XRCC4 and XLF
PE: Plating efficiency
qRT-PCR: Quantitative real-time polymerase chain reaction
RAD51: RAD51 recombinase
RT: Radiotherapy
SF: Surviving fraction
SMI: Small molecule inhibitor
TBS: Tris-buffered saline
XLF: XRCC4-like factor
XRCC4: X-ray repair cross-complementing protein 4
7AAD: 7-Aminoactinomycin D
