# Supplementary material for: Inhibiting NHEJ in HNSCC cell lines by the ligase IV inhibitor SCR130 has limited radiosensitizing effects
Source: Sci Rep. 2025 May 22;15:17871. doi: 10.1038/s41598-025-03159-5 (PMC12098888; doi:10.1038/s41598-025-03159-5)
Supplement: Supplementary file 1 — Supplementary Material 1 [file 41598_2025_3159_MOESM1_ESM.pdf]

Supplementary Figure 1:

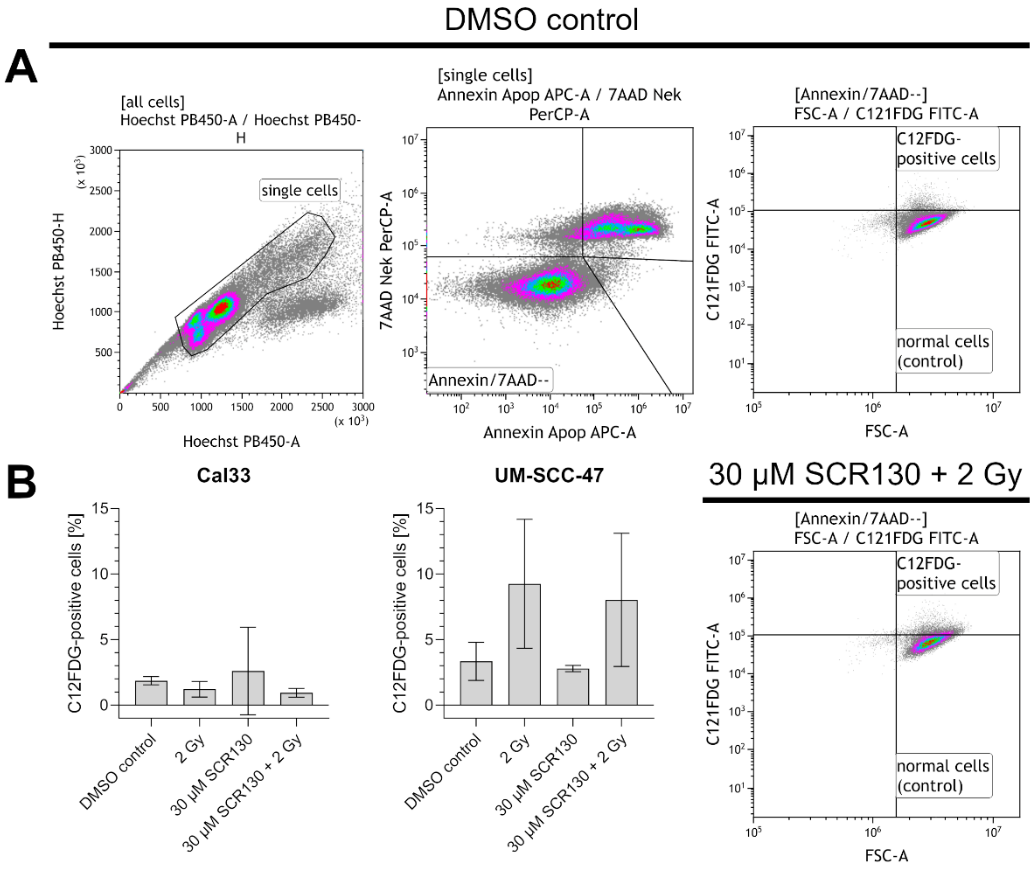

Measurement of C12FDG as a marker of senescent cells after treatment with SCR130 and IR. (A) Gating strategy of C12FDG determination: Single cells were identified using Hoechst signal and living cells using Annexin V and 7AAD signal (Annexin/7AAD--). Only single living cells were analyzed regarding their C12FDG signal. The threshold for a positive C12FDG signal was defined with the DMSO control of every experiment and transferred to the other conditions. Exemplary, C12FDG signal for UM-SCC-47 in the DMSO control and after combined treatment are presented. (B) Cal33 and UM-SCC-47 were treated with 2 Gy IR, 30 µM SCR130 or a corresponding volume of DMSO as vehicle control. For the combined treatment (30 µM SCR130 + 2 Gy) IR was applied 3 h after SCR130 addition. C12FDG positivity was analyzed ten days after seeding. Each value represents mean ± SD (n=4).
